# News and Events of the Week

**Published:** 1911-01-28

**Authors:** 


### News and Events of the Week.

Dr. William Hoffmeister, M.V.O., of Cowes (formerly surgeon to Queen Victoria), who died on March 28 last, aged sixty-seven, left estate valued at £4,080 gross, with net personalty £4,035.

Mr. R. B. D. Acland, K.C., a vice-president of the Hospital Saturday Fund, will take the chair at the annual dinner in the Holborn Restaurant on February 11. The first meeting of the reconstituted Board of Delegates for the year will be held on February 18.

Mr. Frank Hamilton Oswald, M.B., a Dublin University graduate, who left Waterford a few years ago to become house surgeon at Cheltenham Eye, Ear, and Throat Hospital, committed suicide at Cheltenham last Saturday by cutting a vein in the leg.

His Excellency the Earl of Aberdeen, K.T., has consented to act as patron of the twenty-sixth annual Congress of the Royal Sanitary Institute, to be held at Belfast from July 24 till July 29, 1911. The Right Hon. Lord Dunleath, D.L., J.P., will be President of the Congress. The public meeting to inaugurate arrangements for the meeting will be held at the City Hall, Belfast, on Tuesday, January 31.

The German Emperor's collection of academic honours will shortly be enlarged by the bestowal upon him of a medical degree by the German University of Prague, says the *Times* correspondent. The Prague University authorities are stated to have obtained the requisite sanction from the Emperor Francis Joseph. The ceremony will be performed in the Throne Room of the Berlin Schloss by the Rector of the Prague German University, assisted by the senior professors of the Medical Faculty "in their most magnificent attire."

Timothe Bright, Doctor of Phisicke (1550-1615), was for some years resident physician at St. Bartholomew's Hospital. His "Treatise of Melancholy" is said to have influenced Shakespeare as well as Burton, but his most notable achievement was the invention of a system of stenography for which he received a patent from Queen Elizabeth, and he is generally recognised as "The Father of Modern Shorthand." Mr. Elliot Stock, of 62 Paternoster Row, E.C., announces for publication a biography of the learned doctor, written by Mr. W. J. Carlton, whose researches have brought to light many particulars hitherto unknown. The work will be illustrated with photographs and facsimiles.

The annual series of lectures to be delivered at the Royal College of Surgeons will begin on February 1, when Professor F. W. Edridge-Green will deliver the first of two lectures on "Colour Vision and Colour Blindness." The second lecture will be given on February 3. Two lectures will be delivered by Professor W. d'Este Emery on "The Immunity of Reaction in Relation to Surgical Diagnosis" on February 6 and 8, and Professor Benjamin Moore is to give one lecture on "New Views on the Chemical Composition and Mode of Formation of Renal Calculi, and the Metabolism of Calcium in Gout" on February 10. Professor G. Elliot Smith is to give three lectures on "The History of Mummification" on February 13, 15, and 17.

Mr. Acting-Consul Dunn's recent report on the trade of Bangkok affords a striking instance of the way in which considerations of health are sacrificed to appearance. He states: "The disease known as beri-beri has within recent years been occupying the attention of the Government medical authorities in Siam and the adjacent countries, and their investigations tend to show that the consumption of over-milled (polished) white rice is the cause of the disease." White rice is exported from Siam in large quantities, two of the principal markets being Singapore and Hong Kong, and it is pointed out by the Bangkok millers that nothing short of total prohibition of the imports of this quality of rice will put a stop to the trade, the whitening and polishing of the rice being done to meet the demand which exists in these and other markets.

The November number of *Peru To-day* contains an article by R. T. Hird on Sanitary Progress in Peru. The author, who is the municipal sanitary engineer of Callao, states that the Province of Callao has an area of 37 square kilometres, with a population at the last census in 1905 of 34,436. The town occupies 5 square kilometres. It is only since 1870 that energetic measures of sanitation have been enforced, and prior to that the water supply was defective. In 1902 an adequate sewage-disposal system was inaugurated at a cost of £41,200. At the same time the water supply was increased so as to give 30 gallons per head per diem to a population of 50,000, in addition to adequate provision for protection from fire. The quality of the water is kept up to standard by frequent bacteriological examinations. That the result of these measures is beneficial to the health of the community is proved by the health statistics of the town for the past ten years.

